# Supplementary material for: Reading-out task variables as a low-dimensional reconstruction of neural spike trains in single trials
Source: PLoS One. 2019 Oct 17;14(10):e0222649. doi: 10.1371/journal.pone.0222649 (PMC6797168; doi:10.1371/journal.pone.0222649)
Supplement: S1 Fig — (PDF) [file pone.0222649.s001.pdf]

S1 Fig

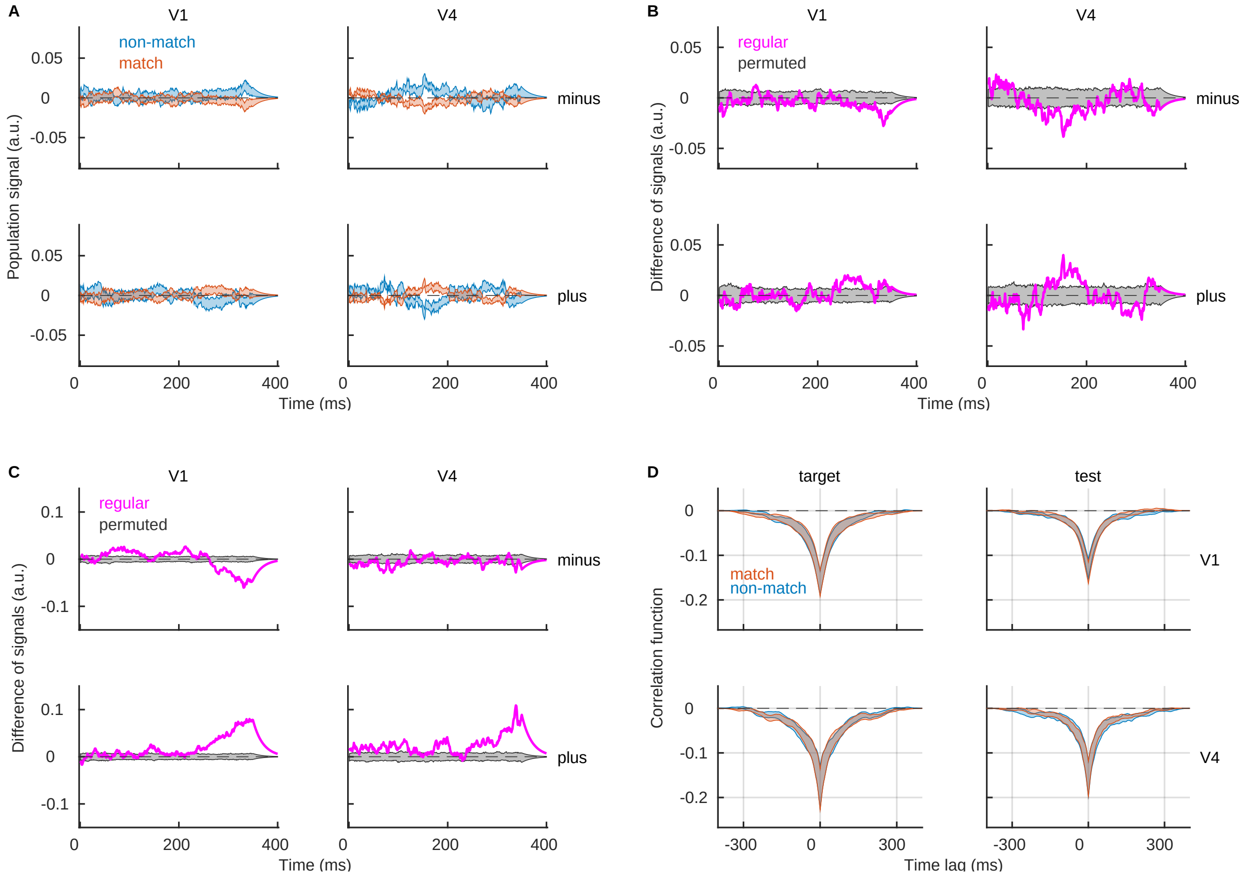

Figure 1. Population signal for neurons with positive and negative weights. (A) Population signal for minus neurons (top) and plus neurons (bottom) during target time window. We show the mean ± SEM across recording sessions in conditions "match" (red) and "non-match" (blue). **(B)** Session-averaged difference of population signals during target. We show the result of the regular model (magenta) and the distribution of results of models with permuted class labels (gray). **(C)** Same as in **(B)**, but for the test time window. **(D)** Correlation function between population signals of plus and minus subpopulations in condition "match" (red) and "non-match" (blue). Parameters: $\lambda = 20^{-1}\ ms$, $nperm = 1000$.
