# Supplementary material for: Reading-out task variables as a low-dimensional reconstruction of neural spike trains in single trials
Source: PLoS One. 2019 Oct 17;14(10):e0222649. doi: 10.1371/journal.pone.0222649 (PMC6797168; doi:10.1371/journal.pone.0222649)
Supplement: S2 Fig — (PDF) [file pone.0222649.s002.pdf]

S2 Fig

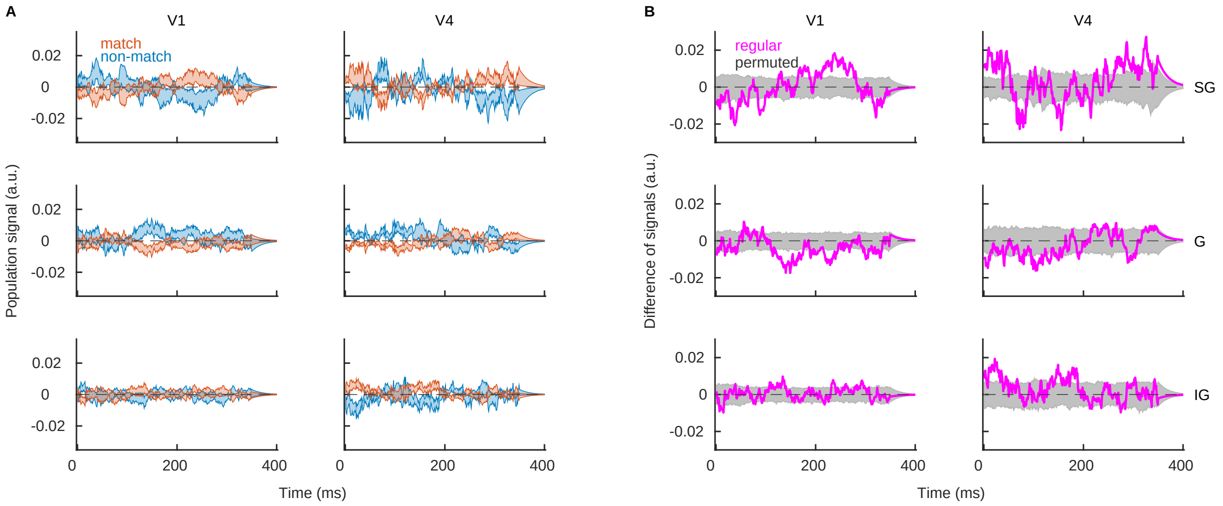

Figure 1. Population signal in cortical layers during the target time window. (A) Population signal in the superficial (top), middle (middle) and deep cortical layers (bottom) in conditions “match” (red) and “non-match” (blue). We show the mean ± SEM in V1 (left) and in V4 (right), where SEM is for the variability across recording sessions . **(B)** Same as in **(A)**, but for the session averaged difference of signals, $x^{diff} = \tilde{x}^{m} - \tilde{x}^{nm}$. We show the result of the regular model (magenta) and the distribution of results for the model with permuted class labels (gray). Parameters: $\lambda = 20^{-1}\ ms$, $nperm = 1000$.
